# The link between the West African Ebola outbreak and health systems in Guinea, Liberia and Sierra Leone: a systematic review

**DOI:** 10.1186/s12992-016-0224-2

**Published:** 2017-01-04

**Authors:** Haitham Shoman, Emilie Karafillakis, Salman Rawaf

**Affiliations:** 1 WHO Collaborating Centre for Public Health Education and Training, Faculty of Medicine, Imperial College London, Charing Cross Campus, Reynolds building, third floor, St Dunstans Road, W6 8RP London, UK; 2 Infectious Disease Epidemiology, London School of Hygiene and Tropical Medicine, London, UK

**Keywords:** Health systems, World Health Organization, Guinea, Liberia, Sierra Leone, Health workforce, Information and research, Health financing, Service delivery, Leadership and governance

## Abstract

**Background:**

An Ebola outbreak started in December 2013 in Guinea and spread to Liberia and Sierra Leone in 2014. The health systems in place in the three countries lacked the infrastructure and the preparation to respond to the outbreak quickly and the World Health Organisation (WHO) declared a public health emergency of international concern on August 8 2014.

**Objective:**

The aim of this study was to determine the effects of health systems’ organisation and performance on the West African Ebola outbreak in Guinea, Liberia and Sierra Leone and lessons learned. The WHO health system building blocks were used to evaluate the performance of the health systems in these countries.

**Methods:**

A systematic review of articles published from inception until July 2015 was conducted following the PRISMA guidelines. Electronic databases including Medline, Embase, Global Health, and the Cochrane library were searched for relevant literature. Grey literature was also searched through Google Scholar and Scopus. Articles were exported and selected based on a set of inclusion and exclusion criteria. Data was then extracted into a spreadsheet and a descriptive analysis was performed. Each study was critically appraised using the Crowe Critical Appraisal Tool. The review was supplemented with expert interviews where participants were identified from reference lists and using the snowball method.

**Findings:**

Thirteen articles were included in the study and six experts from different organisations were interviewed. Findings were analysed based on the WHO health system building blocks. Shortage of health workforce had an important effect on the control of Ebola but also suffered the most from the outbreak. This was followed by information and research, medical products and technologies, health financing and leadership and governance. Poor surveillance and lack of proper communication also contributed to the outbreak. Lack of available funds jeopardised payments and purchase of essential resources and medicines. Leadership and governance had least findings but an overarching consensus that they would have helped prompt response, adequate coordination and management of resources.

**Conclusion:**

Ensuring an adequate and efficient health workforce is of the utmost importance to ensure a strong health system and a quick response to new outbreaks. Adequate service delivery results from a collective success of the other blocks. Health financing and its management is crucial to ensure availability of medical products, fund payments to staff and purchase necessary equipment. However, leadership and governance needs to be rigorously explored on their main defects to control the outbreak.

## Background

Ebola Virus Disease (EVD) was first discovered in 1976, near the Ebola river in the Democratic Republic of Congo (formerly known as Zaire) [1]. More than 25 Ebola outbreaks have been recorded since then, but the West African outbreak which started in 2013 recorded the highest number of deaths compared to all previous outbreaks combined [2]. According to Dr. Margaret Chan, World Health Organisation (WHO) Director General at the time of the outbreak, the 2014–2015 Ebola outbreak was the largest and worst of its kind, jeopardising the fragile health systems and economic stabilities of West Africa [3]. Although it started in December 2013 in a small village in Guinea [4], it was not until August 8 2014 that WHO officially announced Ebola as a Public Health Emergency of International Concern (PHEIC) [5]. As of March 27 2016, total cases were highest in Guinea (3811), Liberia (10,675) and Sierra Leone (14,124), making them the three most affected countries of the region [6].

The failure to contain Ebola has been argued to be a violation of the 2005 International Health Regulations (IHR) which urges member states to detect, assess, report and respond promptly to outbreaks and health emergencies [5]. The magnitude of this outbreak and the speed of Ebola transmission presented a significant threat to international security. The United Nations Security Council therefore unanimously passed a resolution to establish the first ever UN Emergency Health Agency, the United Nations Mission for Emergency Ebola Response (UNMEER) [7]. UNMEER’s responsibilities were transferred to the WHO after it reached its main objective of strengthening surveillance, supporting health workforce and efficiently mobilising resources [8].

The unprecedented spread of Ebola in the region has been explained in many ways and has been linked to factors such as human mobility between the affected countries, behavioural and cultural practices like traditional burials or bush meat consumption, as well as inefficient health systems [9]. Guinea, Liberia, and Sierra Leone have suffered from devastating civil wars, which have had a profound impact on the countries’ health system infrastructure [7]. According to the WHO Health report, they have the lowest human development indexes and among the weakest health system infrastructures in the world [10, 11].

In Guinea, health workforce density (physicians, nurses, midwives, dentists, pharmacists, and psychiatrists) is less than 1.5 per 10,000 population [12], with a total of three hospital beds per 10,000 population [13]. The per capita government expenditure on health is 9 US$ per year [14]. Sierra Leone has a health workforce density of 2.2 per 10,000 population [14] and approximately four hospital beds per 10,000 population [15]. Their per capita government expenditure on health is 12 US$ [14]. In Liberia, the density of the health workforce is less than 3.7 per 10,000 population [14], with around eight hospital beds per 10,000 population [13] and a per capita government expenditure on health of 13 US$ [14]. The public health system infrastructure of the three countries lacked the primary essential elements required to control an outbreak [16], including a strong healthcare workforce [17, 18]. However, the Ebola outbreak also had a significant impact on the already weak health systems and contributed to the decline in the availability of human and physical resources for health.

The aim of this study was to assess existing evidence on the links between the West African Ebola outbreak in Guinea, Liberia and Sierra Leone and the organisation and performance of the countries’ health systems by undertaking a systematic literature review completed by structured expert interviews. Two objectives were developed to achieve this aim: 1) identify effects of the health systems in Guinea, Liberia and Sierra Leone on the management of the Ebola outbreak, and 2) explore the impacts of the Ebola outbreak on the existing health systems in the three countries.

### WHO health system building blocks

The WHO states that “*a well-functioning health system working in harmony is built on having trained and motivated health workers, a well maintained infrastructure, and a reliable supply of medicines and technologies, backed by adequate funding, strong health plans and evidence-based policies*” [19]. These elements have been referred to as the WHO Health System Building Blocks, a framework which will be used in this study to explore the health systems of Guinea, Liberia and Sierra Leone [19–21]. They were selected as a set of internationally agreed health priorities set to create a common understanding of what a health system is and how can it be strengthened [22]. A health system involves all organisations, people and actions whose ultimate priority is to promote, restore or maintain health [22, 23]. The six building blocks are *1) health workforce, 2) health financing, 3) information and research, 4) medical products and technologies, 5) leadership and governance, and 6) service delivery* [19–21].

*Health workforce* include service providers such as physicians, nurses, pharmacists and dentists, health management and support workers [21]. At least 23 physicians, nurses and midwives per 10,000 population are needed for well-functioning primary health care interventions [21]. Their availability should be adequately distributed to offer the best outcomes to the entire population [19–21]. Health workforce should be well qualified, capable, responsive and efficient [19–21].

Strong health systems are adequately *financed* to provide people with the services they need while ensuring they do not suffer from the financial hardships of the inability to pay for their care [19–21]. Health financing is indispensable to maintain and improve human welfare by ensuring workforce employment, availability of medicines and offering promotion and prevention public health programs [21].

A well-functioning *information and research* system ensures the effective and timely collection, analysis, distribution, and communication of information [19–21]. Reliable information is needed for policy development, implementation, governance and regulation, training and health education and for the support of the other five building blocks [21]. Information and research is also important for monitoring and evaluation of diseases and programmes and for early warning of health emergencies [21].

*Medical products (including essential medicines), vaccines and technologies* should be available and accessible to the population [19–21]. They should also be of high quality and efficacy, and scientifically proven to be safe and cost effective [19–21]. Essential medicines are those that satisfy the population’s priority needs [21].

A well *led and governed* health system is one that has vital policy frameworks in place, together with proper stewardship, established partnerships, a respect of regulations, and provision of incentives [19–21]. Leadership and governance are closely linked to accountability [21].

Good *service delivery* is achieved when services are delivered in a timely manner, are cost effective, and safe [21]. They should be of high quality and easily accessible to the entire population, independently from their social status or geographical locations [19–21]. Service delivery also includes person-centred services organised around the patient [21].

## Methods

A very limited number of studies about the West African Ebola outbreak were expected to be found due to the recent occurrence of the outbreak. The systematic literature review was therefore supplemented by expert interviews [24]. The steps undertaken to conduct this study follow the Preferred Reporting Items for Systematic Reviews and Meta-Analyses (PRISMA) statement guidance [25]. The study also used the PRISMA flowchart for study selection [26]. PRISMA was adopted and modified as this study did not assess interventions and included qualitative and observational studies.

### Search strategy

Since this study included qualitative and observational data, the SPIDER tool (Sample – Phenomenon of Interest – Design – Evaluation – Research type) was adopted and modified to formulate the research questions and to establish the inclusion and exclusion criteria [27] (Table 1). Search terms (Medical subject headings (MeSH) and Keywords) were developed to identify articles from the following databases: MEDLINE (1946 to July 15th 2015), Embase Classic + Embase (1947 to July 15th 2015) and Global Health (1973 to 2015 Week 27) (Table 2). No publication date criteria was set for the search in order to have a better understanding of the context of the topic. A grey literature search was also performed in the Cochrane library, Scopus, Google Scholar, and various institutional websites (WHO, Médecins Sans Frontières (MSF) and Centers for Disease Prevention and Control (CDC)). Finally, additional studies or grey literature were searched for in the reference lists of all included articles. Selected articles were exported to Endnote X7 to organise, screen and group them. After all articles were exported, duplicates were removed electronically and manually.

**Table 1:** SPIDER research question formulation

Sample	Guinea, Liberia and Sierra Leone
Phenomenon of Interest	2014/2015 Ebola outbreak
Design	Reviews and reports
Evaluation	Ebola outbreak and health systems organisation and performance
Research type	Qualitative studies

**Table 2:** MEDLINE Database Search

Number	Search	Results
1	(ebola or EVD or ebolavirus or EHF).mp. [mp = title, abstract, original title, name of substance word, subject heading word, keyword heading word, protocol supplementary concept word, rare disease supplementary concept word, unique identifier]	4913
2	Hemorrhagic Fever, Ebola/	1844
3	1 or 2	4913
4	(Sierra leone or liberia or guinea or west* africa).mp. [mp = title, abstract, original title, name of substance word, subject heading word, keyword heading word, protocol supplementary concept word, rare disease supplementary concept word, unique identifier]	163,908
5	Sierra Leone/	853
6	Liberia/	750
7	Guinea/	549
8	Africa, Western/	4467
9	4 or 5 or 6 or 7 or 8	165,741
10	(health* adj3 (workforce or labo?r or personnel or manpower or staff or leadership or govern* or human resource* or financ* or pay* or expenditure or fund* or manag* or admin* or regulat* or technolog* or service* deliver* or information or research* or surveillance)).mp. [mp = title, abstract, original title, name of substance word, subject heading word, keyword heading word, protocol supplementary concept word, rare disease supplementary concept word, unique identifier]	325,886
11	Health Manpower/	11,641
12	Health Expenditures/or Financing, Government/or Financial Management/or Healthcare Financing/or Health Services Accessibility/	100,798
13	“Delivery of Health Care”/	69,493
14	Health Promotion/or Consumer Health Information/or Health Education/or Information Services/	120,760
15	Health Services Research/	32,015
16	“Equipment and Supplies”/	18,766
17	Program Development/or Leadership/or Public Health Administration/or Health Priorities/	75,067
18	Public Health Surveillance/or Population Surveillance/	49,607
19	(Health* system* or WHO building block* or World health organi?ation building block* or surveillance or medical product* or service delivery).mp. [mp = title, abstract, original title, name of substance word, subject heading word, keyword heading word, protocol supplementary concept word, rare disease supplementary concept word, unique identifier]	204,965
20	10 or 11 or 12 or 13 or 14 or 15 or 16 or 17 or 18 or 19	790,837
21	3 and 9 and 20	147

### Study selection (inclusion and exclusion criteria)

The review only included articles from Guinea, Liberia and Sierra Leone, as they recorded the highest numbers of cases and deaths ever resulting from an Ebola outbreak [28]. Studies on Ebola outbreaks before 2014/2015, Ebola outside these three countries, or any other outbreaks, such as haemorrhagic fevers, were excluded. Articles on randomised control trials, vaccines, treatments, clinical picture, disease manifestations, modelling and epidemiological studies, military interventions and commentaries were excluded. Reports from international organisations such as WHO, CDC, and MSF were included. Only papers published in English were considered as translation services were unavailable during this study.

Studies identified with the search strategy, were screened for eligibility through their titles and abstracts according to the predetermined inclusion and exclusion criteria. Studies that met eligibility criteria were then referred for further full text assessment (Table 3).

**Table 3:** Study selection criteria

Inclusion criteria	Exclusion criteria
2014/2015 Ebola outbreak	Ebola outbreaks before 2014
Guinea, Liberia, Sierra Leone	Other countries
Only papers published in English	Any other outbreaks apart from Ebola (like haemorrhagic fevers)
Reports from Internationally recognised organisations (WHO, CDC, MSF)	Articles on randomised control trials, vaccines, treatments, clinical picture, disease manifestations, ethical considerations
Review articles	Non English papers
Articles on the health systems in these three countries	Articles on military and international interventions, modelling and epidemiological analysis
	Opinions, perspectives, editorials and commentaries

### Data extraction and analysis

Data was extracted into a spreadsheet created in Microsoft Excel (Tables 4 and 5) based on the research questions formulated using the SPIDER tool. Extracted data included the study setting, author, title, aims of the study, study design and methods, study area and year, health system building block being discussed, key findings, future recommendations, issues raised during the study, and forms of bias. Studies were analysed and assessed using the Crowe Critical Appraisal Tool (CCAT) [29]. The CCAT has 8 main sections with scores ranging from 0 to 5 giving it a total score of 40 per study [29]. The CCAT was used as it was proven to be valid and reliable to all research designs [30].

**Table 4:** Data extracted and results of all the four studies

Study no.	Author	CCAT Score (/40)	Study design and methods (design & research type)	Title	Aims (phenomenon of interest)	Study area and year (sample)	Objective	Health system building block(s)	Key findings (evaluation)
1	Alexander et al.	16	Review of literature on the challenges and opportunities that led to the Ebola outbreak.	What Factors Might Have Led to the Emergence of Ebola in West Africa?	Review the sociological, ecological and environmental drivers that might have influenced the emergence of Ebola in this region of Africa and its spread throughout the region. The paper also offers recommendations that might support enhanced country level preparedness in Africa.	Guinea, Liberia, Sierra Leone - 2015	*Impacts the Ebola outbreak brought about on the existing health systems in Guinea,*	Health workforce	* Ebola jeopardised the reputation of staff as they were harshly stigmatized and rejected by their communities and families. This made the community turn against seeking health from fear of contracting Ebola from staff. * By September 2014, in the West African outbreak, 10% of who died were health staff. Lack of health staff in hospitals made other staff work longer and harder leading to exhaustion and increased potential for fatal mistakes.
2	Buseh et al.	22	Review. Our approach is guided by the use of literature in peer-reviewed journals on disease burden and health system reforms in developing countries, specifically in sub-Saharan West African countries. Periodicals released by international organizations, including the United Nations Development Program, WHO, United States Agency for International Development, and the World Bank, on global health challenges were also relied on to critically examine the health, socio-political, and economic conditions and to identify the priority and policy areas discussed in this article. We extrapolated from these the studies to critically examine the challenges and opportunities that, if understood and addressed, can effectively contribute to halting the spread of Ebola and potential infectious diseases specific to the West African setting.	The Ebola epidemic in West Africa: challenges, opportunities, and policy priority areas	(a) critically examine the socio-political and economic conditions that created the environment for the Ebola epidemic to occur, (b) identify challenges to and opportunities for the prevention and control of Ebola and future outbreaks, (c) Discuss policy recommendations and priority areas for addressing the Ebola epidemic and future outbreaks in West Africa.	Guinea, Liberia, Sierra Leone - 2015	*Effects the health systems in Guinea, Liberia and Sierra Leone had on the management of the Ebola outbreak*	Medical products and technology/service delivery	* Due to lack of proper transportation and ambulances, access to supportive medical needs and primary care services was compromised. * There was a severe lack of medical equipment (PPE) and supplies. Equipment were used more than once and this led to more infection transmission between health personnel and increase in comorbidities. * There is also a shortage in technologies used for diagnosis and disease management. * There is also poor road networks, lack of electricity, no medical records and poor information and technologies for rapid access to medical services.
								Information and research	* Disease surveillance is very limited in West Africa and health centres face logistical issues on a regular basis. * African traditional healer are the first point of access for health services in rural areas. This happened due to the lack of adequate treatments for patients. *(Clash of Ethno-medicine with Biomedicine)*
								Health workforce	* The three countries are emerging from civil wars and this deprived them from properly trained and competent enough healthcare workers. Prior to Ebola, democracy was still taking place and the countries’ health staff were trying to serve the population’s health needs in-spite the slow economic growth and development of these countries.
								Health financing	Severe shortage of funds for the purchase of supplies and the investment in the infrastructure.
							*Impacts the Ebola outbreak brought about on the existing health systems in Guinea, Liberia and Sierra Leone*	Health workforce	* Staff that were experienced in prevention and treatment of infectious diseases were the frontline victims in this outbreak while they were trying to treat the cases. This caused a deprivation in health workforce in the already weak health system. * Ebola took the motivation of nurses to work and the lives of prominent doctors and nurses depriving the countries from the experienced and dedicated medical teams.
3	Gostin et al.	16	A review and analysis on the health system problems in West Africa. The papers offers recommendation for policy reforms	A retrospective and prospective analysis of the west African Ebola virus disease epidemic: robust national health systems at the foundation and an empowered WHO at the apex	Suggest leadership innovative reforms to transform the worldwide health system into a purposeful organised one.	Guinea, Liberia, Sierra Leone - 2015	*Effects the health systems in Guinea, Liberia and Sierra Leone had on the management of the Ebola outbreak*	Health workforce	There was a huge gap in the health workforce involved in the outbreak control and human resources response required and international action. Apart from international support, there was still shortages in health workforce supply at the Ebola treatment centres despite the Ebola cases rising in December in Sierra Leone. The UN estimated that more than 1000 international staff are still needed.
4	Trad et al.	10	Review of pervious literature on technological approaches to link patients with health care facility. The paper proposes an SMS approach to link patients with health facilities.	Guiding Ebola patients to suitable health facilities: an SMS-based approach	SMS based approach to help map closest health facilities for Ebola patients	Guinea, Liberia, Sierra Leone - 2015	*Effects the health systems in Guinea, Liberia and Sierra Leone had on the management of the Ebola outbreak*	Medical products and technologies	* Patients had the problem of finding the closest health facilities with adequate supplies and resources. They were also confused to whom to report the cases.

**Table 5:** Data extracted and results from all nine reports

Report number	Author	Title	Objective	Building block	Relevant data extracted and results
1	WHO	How Liberia reached zero cases of EVD	Impacts the Ebola outbreak brought about on the existing health systems in Guinea, Liberia and Sierra Leone	Leadership and governance	Leadership and coordination of the country president, health officials and the government was adequate. Presidential advisory committee on EVD was established and introduction of an incident management system helped ensure that resources and capacities were placed when needed. Health officials realised the importance of community engagement and responded accordingly.
				Health financing	This added more supplies, human resources, more treatment beds, increased laboratory capacity, more contact tracing and ensured safe burial teams were deployed. Funding helped installation of transparent walls around treatment centres allowing families and friends to watch what was happening, thus increasing trust. Funding helped increase transportation to treatment facilities. Most of this was international funding.
2	WHO	Ebola virus disease (EVD) in West Africa: an extraordinary epidemic	Effects the health systems in Guinea, Liberia and Sierra Leone had on the management of the Ebola outbreak	Information and research	Limited EVD detection and diagnostics facilities was a reason for the silent progress of Ebola in West Africa.
				Medical products and technologies	Health centres lack resources to implement basic infection prevention measures.
				Health workforce	Limited health workforce was noticed and this added a burden on health centres leading them to more infection transmission and slow response.
			Impacts the Ebola outbreak brought about on the existing health systems in Guinea, Liberia and Sierra Leone	Health workforce	The epidemic was characterised by the high numbers of health care personnel infected by the disease. In the 3 countries, more than 800 contracted the disease and more than 500 died.
3	WHO	Report of the Ebola interim assessment panel	Effects the health systems in Guinea, Liberia and Sierra Leone had on the management of the Ebola outbreak	Information and research	The panel found that there was no data collection, aggregation, analysis or shared in a timely manner and sometimes not at all. There was also lack of proper surveillance.
			Impacts the Ebola outbreak brought about on the existing health systems in Guinea, Liberia and Sierra Leone	Health workforce	There was a noticed short deployment of personnel. This added to the outbreak chaos where there as a constant, rapid turnover of staff and some didn’t have the required capacities. There was a shortage in the coherent human resources management process as some were confused of their exact job description and to whom to report.
4	CDC	A plan for the community event-based surveillance to reduce Ebola transmission – Sierra Leone, 2014–2015	Impacts the Ebola outbreak brought about on the existing health systems in Guinea, Liberia and Sierra Leone	Information and research	Community Event – Based Surveillance (CEBS) system was developed to help strengthen the country’s Ebola surveillance and response capabilities. It is developed to supplement case finding and contact tracing, the core of Ebola surveillance in the West African response. CEBS started in a few low and medium transmission districts and will be deployed to other parts of Sierra Leone.
5	CDC	Use of a nationwide call centre for Ebola response and monitoring during a 3 day house to house campaign – Sierra Leone, September 2014	Impacts the Ebola outbreak brought about on the existing health systems in Guinea, Liberia and Sierra Leone	Information and research	A national Ebola toll free call centre was established in Sierra Leone by the Emergency Operations Centre. This helped to report cases and deaths to public health officials and offers health education messages to the public. This also helps supporting surveillance and alerting any emergencies. Public health officials, then respond promptly delegating tasks to the concerned personnel and start contact tracing and follow up (such as safe transportation of Ebola patients to treatment units or safe burials)
6	CDC	Rapid assessment of Ebola infection prevention and control needs--six districts, Sierra Leone, October 2014	Effects the health systems in Guinea, Liberia and Sierra Leone had on the management of the Ebola outbreak	Medical products and technology.	* There was insufficient PPE supplies, running water, incinerators, chlorine and blood collection supplies all over the three countries. * Due to delays in patient transport and bed availability, home care took place and carers were not trained on ow to deal with cases properly. * There were delays in transporting patients and specimens due to limited availability of ambulances and fuel. The vehicles were not even properly decontaminated. This risks the lives of the teams and the suspected patients. The time between confirming results was long and this jeopardised the process of separation of patients suspected from confirmed in holding centres.
				Health workforce	* Health systems in the three countries lack adequate number of trained and competent staff in IPC. There was also lack of payments in addition to this shortage that lead more staff to leave and this was compounded by the staff fatigue from the burden of having more patients. IPC training wasn’t properly given to staff, ambulance teams and cleaners where as some burial teams and laboratory technicians had some trainings. This made them unable to safely screen or isolate suspected Ebola cases before transport health centres for treatment.
			Impacts the Ebola outbreak brought about on the existing health systems in Guinea, Liberia and Sierra Leone	Information and research	IPC training protocols and programs were developed by the MoH in Sierra Leone. There was also monitoring and evaluation programs for the IPC implementation and quality assurance using IPC metrics. The National Ebola IPC ensures that gaps are identified and responses offered promptly.
7	CDC	Assessment of Ebola virus disease, health care infrastructure, and preparedness – four counties, South-eastern Liberia, August 2014	Effects the health systems in Guinea, Liberia and Sierra Leone had on the management of the Ebola outbreak	Medical products and technologies.	* There was shortage in gloves, hand washing stations, water jugs (in case there was no water stations), PPE (and staff were not trained in using it), soap, bleach, alcohol hand gel and there was no adequate water nor electricity. Bamboo washing stations were created to compensate for this shortage. There was no waste disposal nor isolation facilities. * There was no proper connections in place as internet, phone signals or even radio. * Transportation was also a major challenged for patients and specimens transport.
				Information and research	No surveillance nor training on case investigation, case management, contact tracing, or safe burial practices had been was offered in counties and hospitals.
			Impacts the Ebola outbreak brought about on the existing health systems in Guinea, Liberia and Sierra Leone	Health workforce	Nurses abandoned facilities, staff left Liberia and in some cases, staff were not paid for three months.
8	CDC	Developing an incident management system to support Ebola response - Liberia, July-August 2014	Impacts the Ebola outbreak brought about on the existing health systems in Guinea, Liberia and Sierra Leone	Leadership and governance	Incident Management System was introduced by the MoH and Social Welfare in the early months of the outbreak.
9	CDC	Challenges in responding to the Ebola epidemic - four rural counties, Liberia, August-November 2014	Effects the health systems in Guinea, Liberia and Sierra Leone had on the management of the Ebola outbreak	Medical products and technologies	* There was a lack in proper communication due to shortage in proper telephone coverage and leaders were not able to notify specified health staff on suspected cases, arrange clinical checks or notify each other on any specimen results on time. * Transportation was also a problem as specimens were not transferred on time to laboratories.
				Health workforce	* Health staff in the four counties reported lacking necessary training in case investigation, contact tracing, infection control (including safe burial practices), and health education * Only Grant Bassa had an ambulance team trained in loading and transport of patients and trained staff in case investigation and contact tracing. * There was only one laboratory technician trained in collecting and handling specimens safely in Grand Cape Mount and Grand Bassa. * Sinoe had not technicians at all. * All counties were short on drugs at clinics and PPE and staff were not trained in using it properly.

### Expert interviews

Expert interviews are a relatively new field of study, and differ from qualitative semi-structured or in-depth interviews in that they only aim to gather information (as opposed to data). Smith et al. mentioned how interviews with experts in a topic of interest can help complement a study [31], while Otto-Banaszak et al. mentioned that experts are people who are responsible for the development, implementation or control of solutions, strategies or policies and have authorised access to certain populations or information on certain decision processes [32]. They also have access to insights on topics that are very important and where insufficient information is available [32].

Experts were identified from published literature and using the snowball method. They were selected to include senior academics, professionals from international organisations and advisors to the Ministries of Health (MoH) who have worked on health system strengthening during the Ebola outbreak in either of the three countries.

Questions, based on the study objectives, were developed and reviewed by two health systems experts. Interviews lasted 30 min and were recorded on a digital recorder with prior participant approval. After being informed that the recorded interviews would be deleted once the study was over, experts approved the disclosure of their answers. Results were transcribed manually, and then extracted into an extraction table in Microsoft Excel. The table included names and affiliation of the experts, date and duration of the interviews, method and location of the interview, building blocks discussed, and their personal opinions about the outbreak. The expert interviews and answers gathered are considered as information to supplement this study and not as additional data.

## Results

**Fig. 1:**
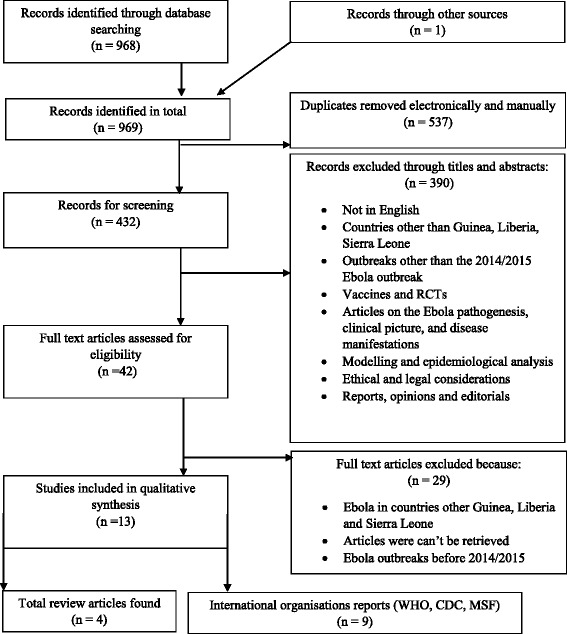
PRISMA Database flow diagram: for the systematic review following the Preferred Reporting Items on Systematic Reviews and Meta-Analysis (PRISMA) flow diagram. A total of 969 articles were found from databases search and external sources. 537 duplicates were removed and 432 were left for screening. 390 were excluded based on titles and abstracts leaving 42 articles for full text assessment for eligibility. Thirteen studies were eventually included after 29 were excluded since they did not meet the eligibility criteria. Of the thirteen articles, four were reviews and 9 were reports

The search strategy yielded a total of 969 articles, out of which 13 articles (four studies and nine reports) were selected for data extraction (Fig. 1) (Table 6). One study scored 55% [33], two scored 40% [34, 35] and one scored 25% on the CCAT quality assessment (Table 7) [36]. Since there was lack of homogeneity of the study designs and no quantitative metrics were found, a meta-analysis was not conducted. Most articles identified, focused on the “medical products and technologies” and “health workforce” WHO building blocks, followed by “information and research”, “health financing”, and “leadership and governance”. No article explored “health system delivery”. Seven experts agreed to take part in the study, out of which one did not send the transcribed version on time for the results to be analysed (Table 8).

**Table 6:** Database search results

Database name	Studies found
MEDLINE	147
EMBASE	284
Global Health	137
Scopus	375
Cochrane	1
Google Scholar	24
Total	968
External report	1
Total	969
After removing duplicates	433

**Table 7:** Crowe Critical Appraisal Tool (results for the four studies)

Study	1. Preliminaries	2. Introduction	3. Design	4. Sampling	5. Data collection	6. Ethical matters	7. Results	8. Discussion	9. Total score (/40)	Percent
	Title - Abstract - Text	Background - Objective	Research design - Exposure - Outcome - Bias	Size - Protocol - Method	Method - Protocol	Participant ethics - Researcher ethics	Analysis, integration - Essential analysis - Outcome	Interpretation - Generalisation - Concluding remarks		
1. Aexander et al.	4	4	2	1	1	0	2	2	16	40
2. Buseh et al.	4	5	2	0	1	0	5	5	22	55
3. Gostin et al.	4	4	3	2	1	0	1	1	16	40
4. Trad et al.	3	4	2	0	0	0	0	1	10	25

**Table 8:** Information extracted and results from the Expert interviews

Name	Date + duration	Affiliation	Communication + Location	Objective	Health System building blocks mentioned	Opinion
1. Gillian McKay	04/08/2015 25 mins 48 s	GOAL International – Behaviour change advisor	Face to face – LSHTM	*Effects the health systems in Guinea, Liberia and Sierra Leone had on the management of the Ebola outbreak*	Health financing	For the most part, the lack of investment in training of healthcare workers, in infrastructure, supply chain management (drugs, PPE, water and electricity), working with the community to increasing their valuing in the health care system is the biggest problem. In SL for example they have the least amount of government funding. Lack of supply chain and training in IPC and general epidemic management would be the most important things.
					Health workforce	They were not well trained and so they were not able to deal with Ebola and lots of them died.
					Information and research	When Ebola came, it wasn’t recognised early and this lead to lots of casualties. This also fits into information and research where it was important to recognise the virus early and allow for contact tracing.
				*Impacts the Ebola outbreak brought about on the existing health systems in Guinea, Liberia and Sierra Leone*	Health workforce	Health workforce! A big problem is the number of healthcare workers who die in Ebola compared to other viral haemorrhagic.
					Service delivery	The shut of schools has led to an increase in teenage pregnancy which added burden to the health systems of the countries. People still do not trust the health systems of these countries. The number of women giving birth decreased in hospitals, increasing maternal mortality. Immunization rates are down and there is potential for other epidemics as a result. HCW decreases as they don’t feel safe to do so.
2. Jimmy Whitworth	30/07/2015 24 mins 23 s	London School of Hygiene and Tropical Medicine - Professor	Face to face – LSHTM	*Effects the health systems in Guinea, Liberia and Sierra Leone had on the management of the Ebola outbreak*	The six blocks (GENERAL)	Simply insufficient money that goes into health systems and that means that they don’t have the staff, finances needed to provide services needed, medical products are only available intermittently, technologies only found in that capital cities, the leadership and governance isn’t very strong. Mostly service delivery isn’t that great. Perhaps in SL it might be better than the others. So it has not been a priority for others.
				*Impacts the Ebola outbreak brought about on the existing health systems in Guinea, Liberia and Sierra Leone*	Service delivery	All general services were not offered adequately as vaccinations, mother and child services. The number of people who died from Malaria are at least as many as those who died from Ebola. Babies who died from diarrhoea were high in numbers as well as from pneumonia. We started seeing epidemics of Measles as these countries were not able to avoid and deal with continuing vaccination programmes
3. Christina Atchison	07/08/2015 27 mins 54 s	NHS England, Imperial College London, Department of Primary Care and Public Health - Clinical Lecturer in Public Health Medicine	Face to face – Imperial College London, Charing Cross Hospital, Department of Primary Care and Public Health	*Effects the health systems in Guinea, Liberia and Sierra Leone had on the management of the Ebola outbreak*	Leadership and governance	Leadership and governance closely linked to planning and financing. Leadership is the overarching. Lack of leadership at the national government level and the organisational leadership in the governmental level and ministries of health
4. Erin Polich	12/08/2015 45 mins 40 s	GOAL International – Emergency and transition coordinator	Skype – London to Freetown, Sierra Leone	*Effects the health systems in Guinea, Liberia and Sierra Leone had on the management of the Ebola outbreak*	Leadership and Governance	Ebola wasn’t mentioned publicly by the president until July of last year and there was a great amount of distrust in SL and they were very fearful of this disease that it was lethal. They were not even sure of it existed and if it did, it wasn’t brought up by the government. There was a lack of understanding by the government and on how communities operate. One of the reasons the outbreak has become so bad and so widespread is that there has been not enough emphasis on the understanding of the distrust of the communities in the health systems and their own governments as well
				*Impacts the Ebola outbreak brought about on the existing health systems in Guinea, Liberia and Sierra Leone*	Service delivery	People were afraid to go the health facilities because people were afraid of being infected if the doctors and nurses are infected and they can’t help themselves. People didn’t want to be transferred to wards as they knew that people walk in as Ebola negative and they walk out being positive. There weren’t enough beds and people were sharing beds and there was lots of contact with vomits, diarrhoea and so on
					Health workforce	These countries are already low in human resources and we have lost hundreds of HCW that we can’t afford to lose. These people have put their heart and soul in defending the lives of other people more than you can imagine. People lost their lives because they were able to be there to care for the Ebola patients. We have lost a huge resource there that would take years and years to build back. One of the really sad things, in SL some of the prominent doctors like Dr. Khan are heroes before Ebola came and were looked up at.
					Health financing	Incredible influx of donor funding in rate that SL has not seen in years which is now available to try to strengthen the government facilities. We see projects that started as IPC projects in hospitals and were now expanded to wider Ebola responses and beyond to build facilities for longer term
					Medical products and technologies	There is now a national IPC unit that exists in the MOH in SL that didn’t exist before to make sure that this doesn’t happen again in the future. There is also the disaster preparedness unit that will be established. If this is funded and coordinated properly, this would be a great resource and we could see a really good thing happening for future outbreaks.
5. David Heymann	24/08/2015 6 mins 22 s	London School of Hygiene and Tropical Medicine/Public Health England/Chatham House - Professor	Phone call – London to Geneva	*Effects the health systems in Guinea, Liberia and Sierra Leone had on the management of the Ebola outbreak*	Health workforce	These countries are recovering from war and many healthcare workers left these countries leaving the workforce in shortage.
				*Impacts the Ebola outbreak brought about on the existing health systems in Guinea, Liberia and Sierra Leone*	Service delivery	The outbreak has affected the countries negatively by shutting down health facilities in some areas and people without signs and symptoms of Ebola had difficulties to get care; some died from other diseases as diarrhoea or malaria, and in some areas of these countries immunisation programmes broke down and there were outbreaks of measles.
6. Chris Lewis	28/08/2015 20 mins 36 s	Department for International Development – Health advisor	Phone call – London to London	*Impacts the Ebola outbreak brought about on the existing health systems in Guinea, Liberia and Sierra Leone*	Health Workforce	Ebola is a crisis that primarily affects healthcare workers and therefore there is a particularly negative impact with the number of healthcare workers that died, those who were sick as well as the psychological impact on healthcare workers.
					Service delivery	There is also the secondary impact on other diseases. It was incredibly difficult to have effective interventions for other health problems when Ebola has affected health facilities

### Health workforce

Five articles argued that a lack of skilled health workforce was a major obstacle in containing the outbreak [33, 35, 37–39]. Two studies mentioned that the lack of workforce was compounded by the civil wars the three countries were emerging from [33, 35]. Furthermore, some of the available healthcare workers lacked the basic infection prevention and control measures (IPC) knowledge [38, 39]. Two experts also supported these findings [40, 41].

Five articles discussed the impact of the Ebola outbreak on existing health workforce [33, 34, 37, 42, 43]. Ebola created an atmosphere of fear among those involved in the outbreak [33, 34, 37, 42, 43], and Alexander et al. indicated that health staff were stigmatised and rejected by communities [34] while an expert expressed that Ebola had a negative psychological impact on healthcare workers [44].

An expert argued that Ebola claimed more lives among healthcare workers than any other viral haemorrhagic fevers [41]. Another expert said “*healthcare workers put their heart and soul in defending the lives of others. We have lost a huge resource there that would take years and years to build back.*” [45]. This weakened an already understaffed healthcare system, and added additional burden on existing healthcare workers, that led to increased exhaustion and fatal mistakes [34]. Buseh et al. ratified this finding recognising that nurses became the frontline of the outbreak which threatened their productivity and decreased their efficiency [33]. A WHO report noted that Ebola also threatened the management of human resources for health in these countries which led to a high turnover rate, and staff not knowing who to report to, and not having the appropriate skills to respond to the outbreak [42].

### Health financing

Buseh et al. mentioned that a lack of adequate funds invested in health system infrastructure and purchase of supplies contributed to the poor management of the Ebola outbreak [33]. According to an expert, the lack of investment in training healthcare workers, infrastructure, supply chain management, and community engagement was the biggest problem [41].

A WHO report found that as a consequence to the outbreak, the international community and the three countries’ governments allocated additional financial support to medical supplies, human resources, beds, laboratory capacity, contact tracing, safe burials and increased transportation services [46]. One expert explained that this outbreak led to an influx of funding for health systems strengthening, which led governments to establish some IPC projects [45].

### Information and research

Four articles stated that shortage of information and research was a considerable problem during the outbreak [33, 37, 42, 43]. Buseh et al. explained that people with Ebola symptoms approached traditional healers, who lacked knowledge on Ebola treatment, resulting in a clash between traditional and modern medicine [33]. Two WHO reports stated there was a severe shortage of surveillance, epidemiological data collection and statistical analysis [37, 42]. Similar views were reflected by experts, explaining that Ebola was not detected early due to a lack of appropriate surveillance methods [41]. All articles stated that health surveys, health system resource tracking, capacity for analysis, synthesis and data validation were lacking [33, 37, 42, 43].

Three CDC reports highlighted the impact of Ebola on the information and research systems of the affected countries. Ebola led to the development of a Community Event Based Surveillance system to help widen and bolster Liberia’s Ebola surveillance and response systems [47]. In Sierra Leone, the Emergency Operations Centre established a toll-free national call centre for Ebola which offers health education messages, flags alerts, and refers people to the appropriate health officials [48]. IPC training protocols, evaluation programs and quality assurance metrics were also developed by Sierra Leone’s MoH [38].

### Medical products and technologies

Studies found that lack of medical supplies, resources, personal protective equipment, electricity and IPC led to an increased rate of infections and poor control of Ebola during the outbreak [33, 36–39, 43]. Delays in testing and diagnosis were also reported due to the paucity in specimen transfer, transportation, ambulances and proper coherent communication methods between health officials, villages and urban areas [38, 39, 43]. One expert argued that Sierra Leone started strengthening information collection and surveillance, with the establishment of a new IPC unit and a disaster preparedness unit [45].

### Leadership and governance

Four articles emphasised the role of leadership in the outbreak without providing specific information [34, 35, 42, 46]. Two experts mentioned that lack of leadership at the national governmental level was the main reason that led to the poor coordination and absence of a prompt response [45, 49]. One expert explained that Ebola was not publicly discussed by the Liberian President until July 2014 [45].

Only two reports noted the impact of Ebola on leadership and governance in Liberia [46, 50]. A presidential advisory committee on Ebola was established and an Incident Management System (IMS) was introduced to ensure capacities and resources were available [46]. After these two initiatives were developed, health officials saw the benefits and the need for community engagement [46].

### Service delivery

No article studied service delivery. However, five experts explained how Ebola impacted the delivery of health services during and after the outbreak [40, 41, 44, 45, 51]. Two experts emphasised that none of the general health services were offered adequately [40, 51]. One expert said: “*Ebola’s burden has jeopardised the services being delivered and led to high numbers of comorbidities and fatalities from malaria. People who died from malaria were twice as much as Ebola. Babies died from diarrhoea and pneumonia*” [51]. Another expert observed that the shutdown of schools led to an increase in teenage pregnancy and said: “*the number of women giving birth in hospitals decreased causing a rise in maternal mortality. Immunization rates fell and other epidemics (as measles) emerged*” [41]. Two experts stated that patients were afraid of seeking treatment due to loss of faith in the medical system; people believed that if healthcare workers were not able to cure themselves, they would not be able to help their patients either [44, 45].

## Discussion

This systematic review, complemented by expert interviews, explored the nature of the link between the West African Ebola outbreak and health systems in Guinea, Liberia and Sierra Leone.

### Health workforce

The results from this study suggest that health workforce had a major effect on the control of the outbreak. In fact, other countries previously affected by Ebola effectively controlled their outbreaks by quickly deploying skilled health staff [9, 16, 52, 53]. Guinea, Liberia and Sierra Leone had not faced outbreaks of such magnitude before and therefore lacked the experience and resources to respond promptly and appropriately [9]. For instance, although Nigeria does not have a highly developed health system, they immediately mobilised their health workforce to prevent Ebola from spreading, for instance including epidemiologists trained in the control of polio [16, 40, 41, 52, 54].

Shortage of healthcare workers is also likely to be due to recent civil wars [16, 55]. Poor working conditions and salaries made healthcare workers flee to other countries when the Ebola outbreak started, which has been seen in other countries during past outbreaks [56, 57]. Surveillance, diagnostic facilities and investigation of cases are important to control an outbreak, but these would be useless without trained health workforce [53]. For this reason, the international community sent foreign staff to support the construction of treatment units, treatment of patients and setting up surveillance systems [5, 7, 53].

Healthcare workers face a particularly high risk of infection and death, as they are in direct contact with symptomatic patients [58–60]. This also creates an indirect impact on other health staff, who are afraid and anxious to face rejection from their families and communities [16, 55, 61, 62]. This led to additional burden, exhaustion, a rapid rate of turnover and poor management of healthcare workers [16, 34, 45, 55, 61–63]. Since nurses were the most involved and connected to the community, it would be useful to train them to disseminate effective messages [33]. Ebola survivors can also be recruited to share their experiences and help control future outbreaks [64].

The WHO Emergency Committee on the 2014 Ebola outbreak in West Africa met throughout the outbreak to share updates and discuss actionable points to control the outbreak in affected countries in accordance with the IHR [65]. Some of the recommendations included that health workforce deployed during an outbreak should be very well trained and qualified. Engagement of community leaders is also very important to reach communities with cultural, religious, or ideological oppositions [65]. Adequate medical care should also be made available for airline personnel and communication should be harmonised in case of symptomatic passengers [65]. Funerals and burials should be conducted by well-trained medical staff to avoid transmission of Ebola [65].

### Information and research & medical products and technologies

Healthcare workers in the three affected countries lacked health education and knowledge to control and respond to the outbreak [41, 66]. Nigeria had an Integrated Disease Surveillance and Response programme and the Nigerian Centre for Diseases Prevention and Control, which conducted epidemiological work, data gathering, surveillance and analysis [52]. It also used innovative technologies to help mapping, identification, investigation, management and follow up of cases [67]. Senegal also had an established “Institut Pasteur laboratory” in Dakar to identify cases and respond promptly [68]. Tambo et al. concluded that having an adequate efficient surveillance response system with early warnings and the capability of determining transmission projections is crucial to monitor and control epidemics [69].

Traditional medicine is highly prevalent in West Africa, which has been known to sometimes conflict with modern medicine [33]. Cultural traditions and a lack of healthcare workers meant that patients sought help from traditional healers, who did not always have the appropriate knowledge to provide adequate advice but also protect themselves from infection [9, 52].

All three countries noted an extreme deficiency in medical supplies, efficient transport systems, communication methods and diagnostic tools [33, 63, 64, 70]. Their collective availability is essential to successfully control Ebola according to Raabea et al. [71]. It is vital to have stronger communication and surveillance, isolation, IPC training, testing protocols, Geographical Information Systems (GIS) and modelling to estimate disease projections [38, 39, 42, 72]. Since Ebola has always affected developing countries, there was little incentive in investing in vaccines and drugs development [55, 58, 73]. However, once the outbreak was declared a PHEIC, pharmaceutical companies started working on the development of various new vaccines [64].

In line with the IHR, the Heads of States should activate their emergency management mechanisms and establish an emergency operation centre as soon as an outbreak starts [65]. Information regarding new cases should be efficiently shared, IPC measures should be deployed and surveillance and diagnostic facilities should be set up [65]. An effective contract tracing and case management system is also required together with an adequate supply of PPE and medical commodities to staff in operation [65].

### Leadership and governance & health financing

Guinea, Liberia and Sierra Leone did not have the adequate policies or IPC strategies in place before the start of the Ebola outbreak [49]. There was a lack of investment made in the infrastructure, training, and in the purchase of needed supplies and drugs [54, 74, 75]. This made it difficult for health officials to respond promptly.

After the outbreak deprived these countries from staff, governments started working with international organisations and research institutions to strengthen capacity and educate the public [76, 77]. Leadership and governance and health financing have an overarching effect on the rest of the health system building blocks. Despite the international support, investment is needed in the countries’ health systems infrastructure for sustainable development. Funding should include adequate payments, support surveillance, purchase of needed supplies and research development [41, 51].

The Emergency Committee advised heads of states of the affected countries to declare a national emergency and inform the public by sharing information on the situation and the importance of community engagement to control the spread of Ebola [65]. The importance of adequate communication between countries should also be emphasised in order to facilitate the mobilisation of necessary services and workforce to control the outbreak [65]. Leadership and resource allocation in this outbreak is of ultimate importance to meet the IHR. It is also important for leaders to ensure that the healthcare workers receive adequate and timely payment for their services as well as appropriate training and education [65].

### Service delivery

Due to a lack of resources, programmes like vaccination campaigns were interrupted, which led to outbreaks of other infectious diseases [16, 40, 54]. Healthcare services for other conditions than Ebola were almost inexistent during the outbreak, which for instance meant pregnant women suffering from high fever were turned down by healthcare workers if they tested negative for Ebola [51, 63]. The stigma attached to healthcare workers also caused patients to avoid seeking treatment for other conditions out of fear [16, 41, 45, 51, 54]. Location of treatment and holding centres was also a problem, with some patients having to travel great distances to reach access any form of care. Improvement in road networks and transport services is important to ensure transportation of patients or specimen on time and improve access to healthcare service delivery. A Short Messaging System has been recommended to map patients with Ebola symptoms to their closest health facilities [36]. In order to comply with IHR, the Emergency Committee advised that treatment centres and diagnostic laboratories should be placed close to places of Ebola transmission and that medical services should also be supplied in a timely manner [65].

### Limitations

There was a very limited number of articles available and the included studies had a low CCAT score and did not evenly assess the health systems comprehensively, as per the WHO health system building blocks. No study offered quantitative metrics to assess the health systems and hence quantitative conclusions could not be drawn. Factors which led to the three countries’ weak health systems prior to the outbreak and those that were not linked to health systems were not explored and could have had an impact on managing the outbreak. This study did not conduct a thorough analysis of the political and economic problems in the three West African countries prior to the outbreak or that contributed to the outbreak, and thus a conclusion cannot be drawn on their links to health systems. The study only looked at articles published in English and therefore might have excluded other relevant articles, particularly French articles from Guinea.

Since this study included expert interviews, some limitations were also associated with the ways experts were identified and invited. A problem with the snowball method used is the exclusion of experts who are not within the same network of the experts being interviewed. This leads to selection bias. The number of experts interviewed was small due to their active involvement in the outbreak. Most experts commented on the situation in Sierra Leone which might have led to an under-representation of issues in Guinea and Liberia. Expert interviews are viewed as and should be treated as information and not data and thus, the results cannot be considered generalizable or representative. Experts based in one of three countries might have also had a biased opinion of the situation in their country which they could have generalised to other countries. Despite searching several databases, there still remains the risk of not capturing articles or reports that were not published and were kept for internal communications between organisations.

### Lessons learned and recommendations

This study offers several implications for practice and research. An already weak health workforce suffered a lot from this outbreak. Since nurses were the most involved and connected to the community, it would be useful to train them so that they can easily disseminate effective messages, for instance for case reporting [33]. The workforce needs to be fully trained, qualified, on standby and familiar with their roles [9, 33, 49]. Employment of Ebola survivors as staff within community and at hospital levels would be helpful in sharing experiences [64]. Despite the international support, investment is needed in the health systems infrastructure to provide sustainable development of the affected countries. Funding should include adequate payments, more surveillance, purchase of needed supplies and research development [41, 51].

Governments should acknowledge the need to respond quickly to outbreaks, learn from successful examples such as those from Nigeria and Uganda, and ensure community engagement is maintained. The IHR needs to be properly adopted and country leaders should ensure in country coordination, collaboration with others and flexible Trade-Related Aspects of Intellectual Property Rights (TRIPS) agreement are in place in case of outbreaks or public health crisis [42, 46]. It is essential to have stronger communication and surveillance, protocols for isolation and testing, modelling to estimate disease projections, GIS and IPC training as they proved to be very efficient in controlling other outbreaks [38, 39, 42, 72]. Improvement in the road networks and transport services is clearly important to ensure transportation of patient or specimen. Medical supplies such as gloves, disinfectants and PPE are needed on a continuous basis [39, 72].

This study does not offer a comprehensive analysis of the health systems of Guinea, Liberia, and Sierra Leone. Further research needs to be undertaken to conduct an extensive health system analysis of the countries before and after Ebola and to assess each building block using quantitative metrics to determine the exact magnitude of problems and the extent of support needed. More research should also focus on community barriers to care, in order to achieve a more patient centred health system.

## Conclusion

Shortage of health workforce was a major challenge in controlling Ebola during the West African outbreak, but was also severely affected by the outbreak. Further research needs to be undertaken to provide a comprehensive analysis of the health systems, including all building blocks, before and after the Ebola outbreak. The importance of community barriers to outbreak control measures also need to be emphasised, to place the patient at the centre of care and ensure a high uptake of interventions by the population.

Ebola is now viewed as an opportunity to rebuild resilient health systems and invest more in capacity building to revive weakened health systems [78]. International attention has also been drawn towards the establishment of an international health systems fund [79] which would help in infrastructure investment. Finally, a strong health system is a result of collective well-functioning building blocks and outbreak control activities should address all of them in order to be successful.

## Abbreviations

CCAT: Crowe Critical Appraisal Tool
CDC: Centres of Disease Control and Prevention
EVD: Ebola Virus Disease
GIS: Geographical Information Systems
IHR: International Health Regulations
IMS: Incident Management System
IPC: Infection Prevention and Control
MeSH: Medical Subject Headings
MoH: Ministry of Health
MSF: Medecins San Frontieres
PHEIC: Public Health Emergency of International Concern
PPE: Personal Protective Equipment
PRISMA: Preferred Reporting Items for Systematic Reviews and Meta-Analyses
SPIDER: Sample, Phenomenon of Interest, Design, Evaluation, Research
UNMEER: United Nations Mission for Emergency Ebola Response
WHO: World Health Organisation

## References

[1] Piot P. No time to lose: a life in pursuit of deadly viruses: WW Norton & Company; 2012

[2] Prevention CfDCa. Outbreaks chronology: Ebola virus disease. https://www.cdc.gov/vhf/ebola/outbreaks/history/chronology.html.

[3] Chan M. Report by the Director-General to the Special Session of the Executive Board on Ebola. World Health Organisation (WHO). 2015

[4] Baize S, Pannetier D, Oestereich L, Rieger T, Koivogui L, Magassouba N (2014). Emergence of Zaire Ebola virus disease in Guinea. N Engl J Med.

[5] Safari SB, Baratloo A, Rouhipour A, Ghelichkhani P, Yousefifard M (2015). Ebola hemorrhagic fever as a public health emergency of international concern: a review article. Emergency.

[6] WHO. Ebola situation report. World Health Organization: 2016.

[7] Boozary ASF, Farmer PE, Jha AK (2014). The Ebola outbreak, fragile health systems, and quality as a cure. JAMA, J Am Med Assoc.

[8] Ki-moon B. Statement by Secretary-General Ban Ki-moon on the transition of UN Ebola emergency respons. United Nations, https://www.un.org/sg/en/content/sg/statement/2015-07-31/statement-secretary-general-ban-ki-moon-transition-un-ebola.

[9] Mbonye AKW, Wamala JF, Nanyunja M, Opio A, Makumbi I, Aceng JR (2014). Ebola viral hemorrhagic disease outbreak in West Africa- lessons from Uganda. Afr Health Sci.

[10] Human Development Index. Report on human development 2014. United Nations Development Programme (UNDP) at: http://hdr.undp.org/en/content/table-1-human-development-index-and-its-components.

[11] WHO. The world health report 2000 - Health systems: improving performance. World Health Organization: 2000.

[12] WHO. Health Workforce - African Health Observatory: World Health Organization - Regional Office for Africa; [25 November 2016]. Available from: http://www.aho.afro.who.int/profiles_information/index.php/Guinea:Health_workforce_-_The_Health_System#Inventaire_et_r.C3.A9partition_des_ressources_humaines_en_sant.C3.A9.

[13] WHO. World health statistics 2014 Report. Global Health Observatory (GHO) data: 2014.

[14] WHO. World health statistics 2015 Report. Global Health Observatory (GHO) data: 2015.

[15] The World Factbook - Hospital bed density [Internet]. Available from: https://www.cia.gov/library/publications/the-world-factbook/fields/2227.html.

[16] Bellizzi S (2014). The current Ebola outbreak: old and new contexts. J Infect Dev Ctries.

[17] Global Health Observatory Data Repository, absolute numbers, data by country. [Internet]. World Health Organization. Available from: http://apps.who.int/gho/data/node.main.A1443?lang=e

[18] Center for Strategic and International Studies. The road to recovery: Rebuilding Liberia’s health system. http://csis.org/files/publication/120822_Downie_RoadtoRecovery_web.pdf

[19] WHO. Health systems: World Health Organization. Available from: http://www.who.int/healthsystems/about/en/

[20] WHO. The WHO Health Systems Framework: World Health Organization. Available from: http://www.wpro.who.int/health_services/health_systems_framework/en/.

[21] WHO. Monitoring the building blocks of health systems: a handbook of indicators and their measurement strategies. World Health Organization Library Cataloguing-in-Publication Data. 2010

[22] WHO (2007). Everybody’s business - strengthening health systems to improve health outcomes : WHO’s framework for action.

[23] WHO. Health Systems Strengthening Glossary. Available from:http://www.who.int/healthsystems/hss_glossary/en/index5.html.

[24] Meuser M, Nagel U, Garz D, Kraimer K (1991). Expertinneninterviews; vielfach erprobt, wenig bedacht. Ein Beitrag zur qualitativen Methodendiskussion. Qualitativ-empirische, Sozialforschung, Konzepte, Methoden, Analysen.

[25] Moher D, Liberati A, Tetzlaff J, Altman D, Group tP (2009). Preferred reporting items for systematic reviews and meta-analyses: the PRISMA statement. Ann Intern Med.

[26] Liberati A, Altman D, Tetzlaff J, Mulrow C, Getzsche P, Ioannidis J (2009). The PRISMA statement for reporting systematic reviews and meta-analyses of studies that evaluate health care interventions: explanation and elaboration. Ann Intern Med.

[27] Cooke A, Smith D, Booth A (2012). Beyond PICO: the SPIDER tool for qualitative evidence synthesis. SAGE Journals.

[28] Centers for Disease Control and Prevention C. 2014 Ebola Outbreak in West Africa - Case Counts. CDC - Ebola (Ebola Virus Disease) - 2014 West Africa Outbreak. 2015; https://www.cdc.gov/vhf/ebola/outbreaks/2014-west-africa/case-counts.html

[29] Crowe M, Sheppard L (2011). A review of critical appraisal toold show they lack rigor: alternative tool structure is proposed. J Clin Epidemiol.

[30] Crowe M, Sheppard L (2011). A general critical appraisal tool: an evaluation of construct validity. Int J Nurs Stud.

[31] Smith V, Devane D, Begley C, Clarke M. Methodology in conducting a systematic review of systematic reviews of healthcare interventions. BMC Med Res Methodol. 2011;11:15. 10.1186/1471-2288-11-15 PMC3039637 21291558

[32] Otto-Banaszak I, Matczak P, Wesseler J, Wechsung F (2011). Different perceptions of adaptation to climate change: a mental model approach applied to the evidence from expert interviews. Springerlink.

[33] Buseh AGS, Stevens PE, Bromberg M, Kelber ST (2015). The Ebola epidemic in West Africa: challenges, opportunities, and policy priority areas. Nurs Outlook.

[34] Alexander KA, Sanderson CE, Marathe M, Lewis BL, Rivers CM, Shaman J, Drake JM, Lofgren E, Dato VM, Eisenberg MC, Eubank S. What factors might have led to the emergence of Ebola in West Africa?. PLoS Negl Trop Dis. 2015;9(6):e0003652. 10.1371/journal.pntd.0003652 PMC4456362 26042592

[35] Gostin LOF, Friedman EA (2015). A retrospective and prospective analysis of the west African Ebola virus disease epidemic: robust national health systems at the foundation and an empowered WHO at the apex. Lancet.

[36] Trad MA, Jurdak R, Rana R. Guiding Ebola patients to suitable health facilities: an SMS-based approach. F1000Research. 2015;4:43. 10.12688/f1000research.6105.1 PMC4358411 25789162

[37] WHO (2015). Ebola virus disease (EVD) in West Africa: an extraordinary epidemic. Wkly Epidemiol Rec.

[38] Pathmanathan IOC, O’Connor KA, Adams ML, Rao CY, Kilmarx PH, Park BJ, Mermin J, Kargbo B, Wurie AH, Clarke KR, Centers for Disease Control Prevention (2014). Rapid assessment of Ebola infection prevention and control needs–six districts, Sierra Leone, October 2014. MMWR Morb Mortal Wkly Rep.

[39] Summers AN, Nyenswah TG, Montgomery JM, Neatherlin J, Tappero JW, T N, M F, M M, Centers for Disease Control Prevention. Challenges in responding to the ebola epidemic - four rural counties, Liberia, August-November 2014. MMWR Morb Mortal Wkly Rep. 2014;63(50):1202–4 PMC5779531 25522089

[40] Heymann D. The link between the 2015/2015 Ebola outbreak and health systems in Guinea., Liberia and Sierra Leone. In: Shoman H, editor. August 12th 2015.

[41] McKay G. The link between the 2015/2015 Ebola outbreak and health systems in Guinea., Liberia and Sierra Leone. In: Shoman H, editor. August 12th 2015. 10.1186/s12992-016-0224-2 PMC5210305 28049495

[42] WHO. Report of the Ebola interim assessment panel. World Health Organization: 2015.

[43] Forrester JD, Pillai SK, Beer KD, Neatherlin J, Massaquoi M, Nyenswah TG, Montgomery JM, De Cock K, Centers for Disease Control and Prevention (2014). Assessment of ebola virus disease, health care infrastructure, and preparedness - four counties, Southeastern Liberia, August 2014. MMWR Morb Mortal Wkly Rep.

[44] Lewis C. The link between the 2014/2015 Ebola outbreak and health systems in Guinea, Liberia and Sierra Leone. Shoman H, editor. 28th August 2015.

[45] Polich E. The link between the 2015/2015 Ebola outbreak and health systems in Guinea, Liberia and Sierra Leone. Shoman H, editor. August 12th 2015. 10.1186/s12992-016-0224-2 PMC5210305 28049495

[46] WHO (2015). How Liberia reached zero cases of Ebola virus disease. Wkly Epidemiol Rec.

[47] Crowe SH, Hertz D, Maenner M, Ratnayake R, Baker P, Lash RR, Klena J, Lee-Kwan SH, Williams C, Jonnie GT, Gorina Y, Anderson A, Saffa G, Carr D, Tuma J, Miller L, Turay A, Belay E, Centers for Disease Control Prevention (2015). A plan for community event-based surveillance to reduce Ebola transmission - Sierra Leone, 2014–2015. MMWR Morb Mortal Wkly Rep.

[48] Miller LAS, Stanger E, Senesi RG, DeLuca N, Dietz P, Hausman L, Kilmarx PH, Mermin J, Centers for Disease Control Prevention (2015). Use of a nationwide call center for Ebola response and monitoring during a 3-day house-to-house campaign - Sierra Leone, September 2014. MMWR Morb Mortal Wkly Rep.

[49] Atchison C. The link between the 2014/2015 Ebola outbreak and health systems in Guinea, Liberia and Sierra Leone. Shoman H, editor. 7th August 2015

[50] Pillai SKN, Nyenswah T, Rouse E, Arwady MA, Forrester JD, Hunter JC, Matanock A, Ayscue P, Monroe B, Schafer IJ, Poblano L, Neatherlin J, Montgomery JM, de Cock KM (2014). Developing an incident management system to support Ebola response - Liberia, July-August 2014. Morb Mortal Wkly Rep.

[51] Whitworth J. The link between the 2014/2015 Ebola outbreak and health systems in Guinea, Liberia and Sierra Leone. Shoman H, editor. London School of Hygiene and Tropical Medicine 30th July 2015.

[52] Oleribe OOS, Salako BL, Ka MM, Akpalu A, McConnochie M, Foster M, Taylor-Robinson SD (2015). Ebola virus disease epidemic in West Africa: lessons learned and issues arising from West African countries. Clin Med.

[53] Gao GFF, Feng Y (2014). On the ground in Sierra Leone. Science.

[54] Etuk EE (2015). Ebola: a West African perspective. J R Coll Physicians Edinb.

[55] Bausch D (2014). Glimmers of hope on the Ebola front. Bull World Health Organ.

[56] Gulland A (2014). More health staff are needed to contain Ebola outbreak, warns WHO. BMJ.

[57] De Frey A (2014). Letters in the time of Ebola. Travel Med Infect Dis.

[58] Chiappelli FB, Bakhordarian A, Thames AD, Du AM, Jan AL, Nahcivan M, Nguyen MT, Sama N, Manfrini E, Piva F, Rocha RM, Maida CA (2015). Ebola: translational science considerations. J Transl Med.

[59] Aylward BBP, Bawo L, Bertherat E, Bilivogui P, Blake I, Brennan R, Briand S, Chakauya JM, Chitala K, Conteh RM, Cori A, Croisier A, Dangou JM, Diallo B, Donnelly CA, Dye C, Eckmanns T, Ferguson NM, Formenty P, Fuhrer C, Fukuda K, Garske T, Gasasira A, Gbanyan S, Graaff P, Heleze E, Jambai A, Jombart T, Kasolo F, Kadiobo AM, Keita S, Kertesz D, Kone M, Lane C, Markoff J, Massaquoi M, Mills H, Mulba JM, Musa E, Myhre J, Nasidi A, Nilles E, Nouvellet P, Nshimirimana D, Nuttall I, Nyenswah T, Olu O, Pendergast S, Perea W, Polonsky J, Riley S, Ronveaux O, Sakoba K, Krishnan RSG, Senga M, Shuaib F, Van Kerkhove MD, Vaz R, Kannangarage NW, Yoti Z (2014). Ebola virus disease in West Africa - the first 9 months of the epidemic and forward projections. N Engl J Med.

[60] Barnes SH, Hussain N, Hogan J, Logan V, Wardrope J (2015). The view from the Ebola Treatment Centre, Makeni, central Sierra Leone. Emerg Med J.

[61] Wiwanitkit V (2014). Unprecedented scale Ebola epidemic in Guinea: what we should know. Asian Pacific Journal of Tropical Biomedicine.

[62] Weyer JG, Grobbelaar A, Blumberg L (2015). Ebola virus disease: history, epidemiology and outbreaks. Curr Infect Dis Rep.

[63] Butler YS (2014). Ebola virus: exposing the inadequacies of public health in Liberia. Mayo Clin Proc.

[64] Brown CA, Arkell P, Rokadiya S (2015). Ebola virus disease: the ‘Black Swan’ in West Africa. Trop Doct.

[65] WHO. Statement on the 1st meeting of the IHR Emergency Committee on the 2014 Ebola outbreak in West Africa World Health Organization: Media centre; 2014. Available from: http://www.who.int/mediacentre/news/statements/2014/ebola-20140808/en/.

[66] Wiwanitkit VT, Tambo E, Ugwu EC, Ngogang JY, Zhou XN (2015). Are surveillance response systems enough to effectively combat and contain the Ebola outbreak?. Infect.

[67] Tom-Aba DO, Olaleye A, Olayinka AT, Nguku P, Waziri N, Adewuyi P, Adeoye O, Oladele S, Adeseye A, Oguntimehin O, Shuaib F (2015). Innovative technological approach to Ebola virus disease outbreak response in Nigeria using the open data Kit and form Hub technology. PLoS One.

[68] WHO. Ebola situation in Senegal remains stable. World Health Organization - Media Centre situation assessment 2014.

[69] Tambo E, Ugwu EC, Ngogang JY. Need of surveillance response systems to combat Ebola outbreaks and other emerging infectious diseases in African countries. Infectious Diseases of Poverty. 2014;3:29. 10.1186/2049-9957-3-29 PMC4130433 25120913

[70] Bosl EDW, Fehling SK, Strecker T, Eickmann M, Diederich U, Otto A, Streubel K, Becker S (2014). Ebola virus disease - handling of personal protective equipment (ppe). [German]. Intensiv- und Notfallbehandlung.

[71] Vanessa NR, Matthias B. Infection control during filoviral hemorrhagic fever outbreaks. Journal of global infectious diseases. 2014;4(1):69–74. 10.4103/0974-777X.93765 PMC3326963 22529631

[72] Klenk HD. Lessons to be learned from the ebolavirus outbreak in West Africa. Emerging microbes & infections. 2014;3(8):e61. 10.1038/emi.2014.68 PMC4150288 26038754

[73] Roca AA, Afolabi MO, Saidu Y, Kampmann B (2015). Ebola: a holistic approach is required to achieve effective management and control. J Allergy Clin Immunol.

[74] Das DG, Guerin PJ, Leroy S, Sayeed AA, Abul Faiz M (2015). The largest Ebola outbreak - what have we learned so far. J Med.

[75] Dhama KM, Malik YS, Malik SV, Singh RK (2015). Ebola from emergence to epidemic: the virus and the disease, global preparedness and perspectives. J Infect Dev Ctries.

[76] Gostin LOW, Waxman HA, Foege W (2015). The president’s national security agenda curtailing Ebola, safeguarding the future. JAMA, J Am Med Assoc.

[77] Griffiths P (2015). Ebola in west Africa: the end of the first year. Rev Med Virol.

[78] Kieny MPE, Evans DB, Schmets G, Kadandale S (2014). Health-system resilience: reflections on the Ebola crisis in western Africa. Bull World Health Organ.

[79] Gostin LO (2014). Ebola: towards an international health systems fund. Lancet.

